# Contrafreeloading and its influencing factors in budgerigars (*Melopsittacus undulatus*): Implications for their feeding and welfare

**DOI:** 10.1017/awf.2025.15

**Published:** 2025-03-20

**Authors:** Yue Tao, Yu-Ting Zhu, Hui Li, Qi-Xin Zhang, Yong Zhu

**Affiliations:** School of Biological and Food Engineering, Hefei Normal University, Hefei 230601, China

**Keywords:** animal welfare, contrafreeloading, effort required, food deprivation, *Melopsittacus undulatus*, pre-training

## Abstract

Contrafreeloading (CFL) refers to animals’ tendency to prefer obtaining food through effort rather than accessing food that is freely available. Researchers have proposed various hypotheses to explain this intriguing phenomenon, but few studies have provided a comprehensive analysis of the factors influencing this behaviour. In this study, we observed the choice of alternative food containers in budgerigars (*Melopsittacus undulatus*) to investigate their CFL tendencies and the effects of pre-training, food deprivation, and effort required on the CFL tasks. The results showed that budgerigars did not exhibit significant difference in their first choices or the time interacting with less challenging versus more challenging food containers. Moreover, when evaluating each budgerigar’s CFL level, only half of them were identified as strong contrafreeloaders. Thus, we suggest that budgerigars exhibit an intermediate CFL level that lies somewhere between a strong tendency and the absence of such behaviour. Furthermore, we also found that food-deprived budgerigars tended to select less challenging food containers, and pre-trained budgerigars were more likely to choose highly challenging food containers than moderately challenging food containers, which means that the requirement of only a reasonable effort (access to food from moderately challenging food containers in this study) and the experience of pre-training act to enhance their CFL levels, whereas the requirement of greater effort and the experience of food deprivation act to decrease their CFL levels. Studying animal CFL can help understand why animals choose to expend effort to obtain food rather than accessing it for free, and it also has implications for setting feeding environments to enhance the animal welfare of captive and domesticated animals.

## Introduction

Optimal foraging theory suggests that animals prefer to choose food that is high in energy while minimising effort (Charnov & Orians 1973; Pyke *et al.* 1977; Stephens & Krebs 1986). Similarly, standard learning theory argues that subjects learn behaviour from maximising rewards while minimising costs (Skinner 1938; Hull 1943). These theories predict that when animals choose between two tasks to get the same food reward, they will choose the easier one. However, many studies have shown that animals prefer to work for their food rather than take free food when faced with an equal amount of food resources. This phenomenon, which contradicts optimal foraging and learning theories, is known as contrafreeloading (CFL) (Jensen 1963; Inglis *et al.* 1997).

CFL has been widely reported in laboratory, captive, and domesticated animals, such as laboratory rats (*Rattus norvegicus*; Inglis *et al.* 1997), laboratory pigeons (*Columba livia*; Neuringer 1969), captive maned wolves (*Chrysocyon brachyurus*; da Silva Vasconcellos *et al.* 2012), captive rhesus macaques (*Macaca mulatta*; Reinhardt 1994), red jungle fowl (*Gallus gallus*; Lindqvist & Jensen 2008), and pigs (*Sus scrofa*; De Jonge *et al.* 2008). Some wild animals in captivity also show a tendency for CFL. For example, McGowan *et al.* (2010) found that captive wild grizzly bears (*Ursus arctos horribilis*) spent more time opening food boxes to get food rather than taking free food. CFL has also been reported in humans. Tarte (1981) found that young people preferred to press a lever to receive candy or cash rewards rather than take them directly. Meanwhile, various researchers have explored this behaviour from different perspectives based on the animals’ living environment and physiological characteristics, aiming to provide reasonable explanations for this phenomenon.

Inglis *et al.* (1997) summarised five possible explanations for this seemingly counterintuitive behaviour. Prior training theory suggests that the training process to obtain food serves as a stimulus and becomes a secondary reinforcer, resulting in animals preferring more challenging foraging behaviour even when free food is available (Alferink *et al.* 1973). Neophobia theory suggests that the training for a task causes animals to fear the novel free food provided (Mitchell & White 1977). Stimulus change theory proposes that any form of sensory change is beneficial, and animals will seek to alter their environment by searching for food, creating novelty (Osborne & Shelby 1975). Information primacy theory argues that working for food provides useful information for future foraging (Singh 1970; Inglis & Ferguson 1986). Self-reinforcing theory suggests that the effort animals put into obtaining food is self-satisfying (Jensen 1963).

From the above explanations, several potential factors might affect CFL levels. First, according to prior training theory and neophobia theory, foraging training may affect animals’ CFL levels. The training process can serve as a stimulus reinforcer or make animals fear free food, leading to higher CFL levels. However, few experimental studies have compared the impact of foraging training on CFL levels. Second, according to information primacy theory, working for food provides useful information for future foraging. However, when the motivation to gather information is replaced by a stronger feeding motivation (such as hunger), information-gathering behaviour decreases (Inglis & Ferguson 1986; Lindqvist *et al.* 2002). This suggests that animals may show different levels of CFL relative to different food deprivation levels. Finally, self-reinforcing theory predicts that the effort required to obtain food can also affect the level of CFL. On the one hand, animals gain satisfaction from the effort of obtaining food, increasing their motivation to persist in challenging foraging tasks (Sasson-Yenor & Powell 2019). On the other hand, if the difficulty level is too high and animals cannot gain satisfaction from overcoming it, they will stop foraging, leading to the disappearance of CFL (Carder & Berkowitz 1970).

Budgerigars (*Melopsittacus undulatus*) are small, climbing birds belonging to the order Psittaciformes, family Psittacidae (BirdLife International 2012), and genus *Melopsittacus.* They are common subjects in animal cognition research. Previous studies have shown that trained budgerigars can complete complex tasks to obtain food, and their problem-solving ability is related to their exploratory behaviour (Chen *et al.* 2019). Therefore, budgerigars are ideal subjects for studying CFL and its influencing factors. The aims of this study were: (1) to assess the existence of CFL in budgerigars by investigating their feeder selection between two food containers (free food container and moderately challenging food container; free food container and highly challenging food container; moderately food container and highly challenging food container); (2) to investigate the potential factors affecting budgerigars’ feeder choices by dividing subjects into two groups: trained and untrained; moreover, all subjects were treated with three levels of food deprivation in order: no food deprivation, moderate food deprivation, and high food deprivation. Thus, our study aimed to analyse the effects of prior training, food deprivation, and effort required on the level of CFL. Studies have shown CFL to be correlated with foraging styles, and budgerigars used extended search to obtain food, similar to pigeons, rats, and gerbils (*Meriones unguiculatus*; Inglis *et al.* 1997). Furthermore, because prior training theory suggests training would serve as a secondary reinforcement and neophobia theory would cause animals to fear the free food, we predicted that budgerigars would exhibit CFL, preferring to select the challenging food containers rather than those with free food. Finally, research indicates that increased food deprivation leads to a reduction in CFL tendencies (Inglis & Ferguson 1986), we predict that budgerigars will exhibit higher CFL levels when not subjected to food deprivation. Conversely, high levels of food deprivation are expected to result in lower CFL levels.

## Materials and methods

### Ethical approval

All the procedures described in this article were approved by the Institutional Committee for Animal Care and Use at Hefei Normal University (see Supplementary material).

### Subjects and apparatus

#### Subjects

This study involved 12 adult budgerigars (six males and six females). All budgerigars were procured from the Hefei Yufeng Market, Yaohai District, China. Healthy and active budgerigars were selected, and none had prior experience with foraging task training. The budgerigars were housed in a well-ventilated and well-lit animal room with a natural light cycle. Four birds were placed in each wire cage, with *ad libitum* access to food (dry, shelled grains) and water. Researchers identified individuals based on external characteristics such as size and feather colour.

#### Apparatus

**Figure 1.**
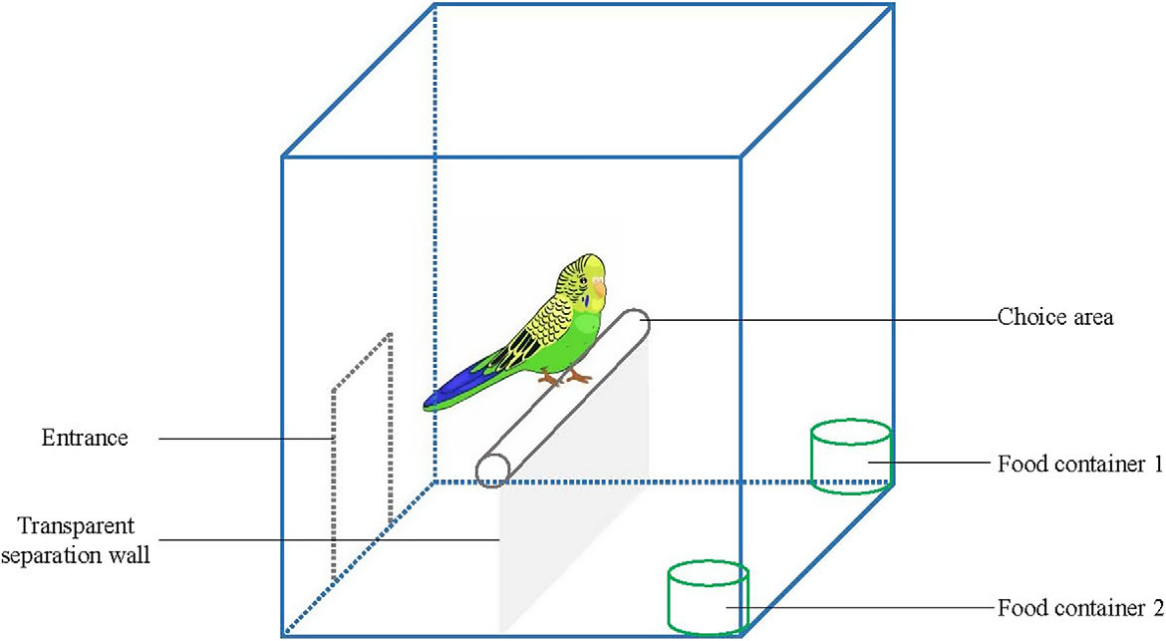
Birdcage used in the experimental set-up to test contrafreeloading (CFL) in budgerigars (n = 12). Image is to scale.

We conducted the experiments in a birdcage (32.5 cm × 30.5 cm × 34 cm; length × width × height), which included a choice area and two food containers (one being less challenging and the other more challenging in terms of obtaining food) (Figure 1). The budgerigars entered the cage via an entrance into an adaptation buffer area between the entrance and a transparent partition (made of glass). This partition prevented agitated animals from entering the food area and affecting the accuracy of the experiment while allowing the birds to observe the food area. When the budgerigars reached the perch, they could observe the two food containers (made of transparent plastic) and make choices based on the foraging challenge.

Three devices were set for animals to obtain food:

- Free food container (Free) – the top of the food container was uncovered, allowing the budgerigars to access the food freely;
- Moderately challenging food container (MC) – the food in the container was covered with wood-shavings, requiring the budgerigars to use their claws or beaks to move the shavings aside to access the food; and
- Highly challenging food container (HC) – the food in the container was covered with wood-shavings, and the top was sealed with plastic wrap (weak enough to be pierced by the beaks of the budgerigars). The budgerigars needed to peck open the plastic wrap and move the shavings aside to access the food.

### Pre-experiment

#### Habituation

The budgerigars were placed in the experimental preparation room prior to the onset of the formal experiment, preventing the researcher’s actions, sounds, and experimental apparatus from unsettling the animals. Researchers fed the birds by hand and placed the required experimental apparatus around them, allowing the budgerigars to become familiar with the experimental environment. As the budgerigars had been artificially raised (by humans), it only took four days for them to no longer display any signs of fearful behaviour and move freely throughout the cage, thereby indicating habituation (Bean *et al.* 1999). For the duration of the study, only our study group’s researchers were allowed access to the room housing the birds.

#### Pre-training

We selected six budgerigars (three males and three females) for pre-training. For each bird, we provided two food containers, one allowed free access to food, and the other was an MC/HC container. The birds need to use both containers to get their 4-h food rations (normal feeding cycles). Each container had the same amount of food, and both together provided the 4-h food ration, encouraging birds to complete the tasks, and meanwhile preventing the influence of stimulus reinforcement. First, for the Free/MC, the budgerigars could see the covered MC food through the transparent plastic food container. If, initially, budgerigars were unable to obtain the food provided (dry, shelled grains) researchers would remove the wood-shavings to encourage the budgerigars to see and eat the food from the top. Once they had successfully obtained the food from MC, researchers replaced the wood-shavings and observed whether the budgerigars could remove the shavings to eat. This was repeated until the budgerigars were able to effectively remove the wood-shavings and consume the food. Second, for the Free/HC, researchers pierced the plastic wrap covering of HC to encourage the budgerigars to pierce the wrap and move the wood-shavings to eat, and the unpierced plastic wrap was presented until the budgerigars could pierce the wrap and move the shavings to eat. Training ended when all six budgerigars could complete these two steps and successfully obtain the food at least three times. These pre-training procedures lasted seven days.

#### Procedure

To prevent interference with sex factors, six males and six females were utilised in our experiment. Before the formal experiment, researchers identified individual budgerigars based on size, feather colour, and specific markings. The experiments were conducted between 0900h and 1500h to avoid any influence of natural light changes. Each budgerigar was placed in a 32.5 cm × 30.5 cm × 34 cm cage for the duration of the experiment. We conducted 10-min videotaped trials, randomly alternating the food containers between the left and right sides to prevent any potential bias caused by the birds’ side preferences.

Six budgerigars (three males and three females) were pre-trained, while the others were not. Given that budgerigars’ feeding cycles are generally 3–5 h, we created three food deprivation levels: no deprivation (NDP, free feeding); moderate deprivation (MDP, 4 h without food); and high deprivation (HDP, 8 h without food) (Bean *et al.* 1999). All birds underwent these three deprivation levels. For each bird, we first tested their selection under no deprivation (NDP) level, and then the moderate deprivation (MDP) level on the second day. On the fourth day (one day was provided for adjustment), we tested their selection under a high deprivation (HDP) level. Two or more days later, another series of trials were started as above, i.e. six trials were conducted for each deprivation level.

For each trial, we recorded the budgerigars’ ID, sex, training status, and food deprivation levels, placing them in the experimental cage. Once the budgerigars were freely moving in the cage without showing any visible signs of stress, their food choices were observed and recorded. The study compared choices: (1) between Free and MC; (2) between Free and HC; and (3) between MC and HC. We determined container preferences by the first choice and the proportion of time spent at the more challenging food containers (Sasson-Yenor & Powell 2019). The proportion of time spent at more challenging tasks was calculated as follows:

time spent at the more challenging food containers/(time spent at the less challenging food containers + time spent at the more challenging food containers).

#### Data analysis

The study first compared the proportions of the first choice for budgerigars with NDP. Additionally, we used paired *t*-tests to compare the time spent choosing more challenging versus less challenging food containers for budgerigars with NDP. Osborne (1977) proposed that if the proportion of choosing challenging food containers exceeded 50%, the subjects were considered to show CFL tendencies (Osborne 1977). However, as optimal foraging theory and standard learning theory predict a total preference for free food, any deviation from such an outcome should be explained by other theories (Inglis *et al.* 1997; da Silva Vasconcellos *et al.* 2012). As a result of such controversies, here the decision was taken to classify the budgerigars’ CFL tendencies into four levels based upon the proportion of time spent at more challenging food containers: strong (> 0.5); moderate (0.3–0.5); weak (0.1–0.3); and null (< 0.1) (Delgado *et al.* 2022).

To analyse the factors that affected CFL levels, three generalised linear mixed models (GLMMs) were constructed using the binomial error structure and logit-link function in R 3.6.3. The dependent variables were the first choice of less challenging and more challenging food containers. Prior training and food deprivation levels were fixed factors, while individual identity and sex were random variables. The GLMM analysis was performed using the glmer function from the lmerTest package in R (Bates *et al.* 2012). Another three GLMMs with Gaussian error structure and identity link function were used to analyse the proportion of time spent at more challenging food containers, with fixed factors and random variables as above. The GLMM analyses were performed using the lmer function from the lmerTest package in R (Bates *et al.* 2012). All data were analysed with the significance level set at 0.05 (two-tailed).

## Results

A total of 216 trials were conducted, with 18 trials per individual in this study. Among them, the budgerigars did not choose in 21 trials, and these instances occurred 16 times under conditions with NDP, five times under conditions with MDP, and never under conditions with HDP. In the remaining 195 trials, the less challenging food containers were chosen first 111 times while the more challenging containers were chosen first 84 times. Table 1 shows the detailed information regarding specific choices. Primarily, for the trials including HC food containers (n = 144), the pre-trained budgerigars spent time at the HC food containers in 62 trials and failed to access food in 29 trials. The untrained budgerigars spent time at the HC food containers in 37 trials and failed to access food in 27 trials.

**Table 1.** Proportion of time each budgerigar (n = 12) chose the challenging food containers in each trial of the study. The larger the value, the higher the proportion of time budgerigars chose the more challenging food container. Numbers in bold indicate that the more challenging food container was the first choice. Blank means no choice

ID	Pre-trained	Sex	Trial 1 NDP(Free vs MC)		Trial 2 MDP(Free vs MC)		Trial 3 HDP(Free vs MC)		Trial 4 NDP(Free vs HC)		Trial 5 MDP(Free vs HC)		Trial 6 HDP(Free vs HC)		Trial 7 NDP(MC vs HC)		Trial 8 MDP(MC vs HC)		Trial 9 HDP(MC vs HC)	
			L	R	L	R	L	R	L	R	L	R	L	R	L	R	L	R	L	R
BNI	Yes	Male	**1.00**	**0.84**	**0.33**	**0.84**	0.52	0.02	**0.99**	0.88	**0.18**	0.56	**1.00**	0.91	**1.00**	0.00	**0.92**	**0.99**	**0.03**	0.05
QFB	Yes	Female	**1.00**	**1.00**	0.00	**1.00**	**1.00**	0.28	0.30	0.58	0.01	0.39	**0.96**	0.46	**0.42**	0.99	0.21	0.66	0.70	0.39
LDD	Yes	Female	**0.89**	0.62	0.08	0.62	0.00	0.74	**0.29**	0.80	**0.12**	**0.08**	**0.01**	0.65	**0.95**	0.96	**0.54**	**0.63**	0.78	0.04
LXD	Yes	Female	0.00	0.00	0.53	0.00	**0.72**	0.30	**1.00**	0.06	**1.00**	0.00	0.00	0.00	0.81	**1.00**	0.00	**0.05**	0.00	0.01
HNI	Yes	Male	**1.00**		**1.00**		**1.00**	**1.00**	**0.57**	**1.00**	**1.00**	**0.50**	**1.00**	**1.00**	**0.97**	**1.00**	0.32	**1.00**	**1.00**	0.46
QDD	Yes	Male	0.41	**1.00**	0.00	**1.00**	**0.66**	**1.00**	**0.31**	0.00	0.11	0.00	0.00	0.00	0.00	0.03	0.00	**0.02**	**1.00**	**0.20**
HCN	No	Female			**0.22**	0.00	**0.30**	0.85				0.00	0.00	0.04		0.00	0.07	0.00	0.00	**0.03**
LDW	No	Female	0.00	**0.35**	0.02	0.64	0.40	**0.01**	0.25			**0.17**	0.00	0.46	0.04	**1.00**	0.05	**0.03**	0.13	**0.40**
QDA	No	Female		0.92	**0.21**	0.43	**0.90**	0.65	**1.00**	0.00	0.14	0.91	0.05	0.09		**0.47**	**0.50**	0.83	0.23	0.00
QSD	No	Male					**1.00**	0.97			0.00	0.00	0.00	0.00			0.00	**0.01**	0.00	0.00
QXD	No	Male	**1.00**	**1.00**	**0.92**	**1.00**	0.00	1.00	0.47	**1.00**	**0.32**	0.02	0.00	0.23	**1.00**	**0.19**	**0.99**	**0.01**	0.00	0.00
LCW	No	Male		**1.00**	0.54	**1.00**	0.04	0.02	**1.00**	**0.01**	0.04	0.00	**0.01**	0.00	0.00	0.00	0.09	0.07	0.00	0.00

ID: Three-letter identification of each budgerigar. NDP: no food deprivation; MDP: moderate food deprivation; HDP: high food deprivation. Free: freely accessible food container; MC: moderately challenging food container; HC: highly challenging food container; L (left) and R (right) indicate the side of the more challenging food container in a trial.

### Budgerigars’ CFL tendencies

Given that the identification of CFL tendency is based upon the choices made by animals not subject to food deprivation, this study compared the first choice and the time proportion of the food containers chosen by the budgerigars with NDP. In the 56 experiments without food deprivation, the budgerigars chose the less challenging food containers first 25 times and the more challenging food containers 31 times (binomial test; *P* = 0.504). Additionally, a paired *t*-test showed no significant difference in the time spent between less challenging and more challenging food containers (*t* = 0.982, n = 56; *P* = 0.330). Further, we calculated each budgerigar’s CFL level and based on these results, we would classify half of the budgerigars (n = 6) as strong contrafreeloaders (CFL levels: 0.62–0.98), three budgerigars as moderate (CFL levels: 0.33–0.46) and one budgerigar as weak (0.21) contrafreeloaders, and one budgerigar as freeloader (CFL level: 0.0). One bird did not choose.

### Factors affecting budgerigars’ first choice of food containers

For the trials set on the first choice, out of 62 first choices between MC and Free food containers, the budgerigars chose the Free food containers 30 times and the MC food containers 32 times. Out of 65 first choices between HC and Free food containers, the budgerigars chose the Free food containers 41 times and the HC food containers 24 times. Out of 68 first choices between HC and MC food containers, the budgerigars chose the MC food containers 40 times and the HC food containers 28 times. We used GLMM binomial regression to assess the factors affecting the budgerigars’ first choice for food containers. The results showed that pre-training (Yes/No) and food deprivation (MDP vs NDP, HDP vs NDP) did not significantly affect the budgerigars’ first choice between the less challenging and more challenging food containers (see Table 2).

**Table 2.** GLMM binomial regression results for the factors on the first choice made by budgerigars (n = 12) in contrafreeloading (CFL) tests. Estimates, SE, Z value, and P-values for the three GLMMs run to test the influence of pre-training and food deprivation on first choice between two food containers: MC vs Free, HC vs Free, and HC vs MC

Independent variable	MC vs Free (n = 62)				HC vs Free (n = 65)				HC vs MC (n = 68)			
	Estimate	SE	Z value	*P-*value	Estimate	SE	Z value	*P-*value	Estimate	SE	Z value	*P-*value
Intercept	0.6340	0.6857	0.925	0.355	−0.6618	0.7351	−0.900	0.3680	−0.3241	0.5627	−0.576	0.565
Pre-training: Yes	0.0686	0.6387	0.107	0.914	1.1636	0.7851	1.482	0.1383	0.5352	0.5399	0.991	0.322
Food deprivation (MDP vs NDP)	−0.7471	0.7049	−1.060	0.289	−0.6512	0.7042	−0.925	0.3551	0.0565	0.6177	0.091	0.927
Food deprivation (HDP vs NDP)	−0.8490	0.6962	−1.220	0.223	−1.2334	0.7302	−1.689	0.0912	−1.0730	0.6624	−1.620	0.105

Free: free to access food container; MC: Moderately challenging food container; HC: Highly challenging food container.

NDP: no deprivation; MDP: moderate deprivation; HDP: high deprivation.

*P-*values < 0.05 are indicated in bold

### Factors affecting proportion of time at more challenging food container

We used GLMM Gaussian regression to assess the factors affecting the budgerigars’ proportion of time spent at more challenging food containers. The results in Table 3 (MC vs Free) showed that both pre-training (Yes/No) and food deprivation (MDP vs NDP, HDP vs NDP) did not have a significant effect on budgerigars’ proportion of time spent at challenging containers. However, for the trials between HC and Free food containers, the results (Table 3; HC vs Free) show that budgerigars with MDP and HDP both tend to select the Free food containers rather than highly challenging food containers while pre-training (Yes/No) did not significantly affect the proportion of time spent at highly challenging food containers. Furthermore, the GLMM results for the trials between MC and HC food containers showed that pre-trained budgerigars were more likely to select HC food containers, and budgerigars with HDP spent a greater proportion of time at the MC than HC food containers.

**Table 3.** GLMM gaussian regression results for the factors on proportion of time spent at more challenging food container by budgerigars (n = 12) in contrafreeloading (CFL) tests. Estimates, SE, t value, and P-values for the three GLMMs run to test the influence of pre-training, and food deprivation on proportion of time spent on the more challenging of two food containers: MC vs Free, HC vs Free, and HC vs MC

Independent variable	MC vs Free (n = 62)				HC vs Free (n = 65)				HC vs MC (n = 68)			
	Estimate	SE	t value	*P-*value	Estimate	SE	t value	*P-*value	Estimate	SE	t value	*P-*value
Intercept	0.7117	0.1429	4.982	0.002	0.3852	0.1180	3.265	0.003	0.3609	0.1075	3.359	0.002
Pre-training: Yes	0.0261	0.1428	0.183	0.859	0.2419	0.1330	1.818	0.097	0.2948	0.1150	2.563	**0.027**
Food deprivation (MDP vs NDP)	−0.2040	0.1186	−1.720	0.092	−0.2673	0.1006	−2.657	**0.010**	−0.1756	0.1033	−1.700	0.094
Food deprivation (HDP vs NDP)	−0.1669	0.1163	−1.435	0.157	−0.2200	0.0994	−2.213	**0.031**	−0.2818	0.1033	−2.727	**0.009**

Free: free to access food container; MC: Moderately challenging food container; HC: Highly challenging food container.

NDP: no deprivation; MDP: moderate deprivation; HDP: high deprivation.

*P-*values < 0.05 are indicated in bold

## Discussion

This study represents the first investigation into contrafreeloading behaviour in budgerigars. The results revealed that, at the group level, budgerigars did not exhibit a statistically significant preference for more challenging food containers. Moreover, only half of the budgerigars exhibited strong CFL levels, and one was even classified as freeloader. Additionally, when examining the influencing factors of CFL levels, we found that pre-training and food deprivation had no significant effect on budgerigars’ first choice between less challenging and more challenging food containers. While food deprivation, especially the HDP, significantly decreased the budgerigars’ proportion of time spent at HC food containers in the trials ‘Free vs HC’ and ‘MC vs HC’, and pre-training increased their proportion of time spent at HC food containers in the trials ‘MC vs HC.’ Our results indicated that budgerigars exhibit a CFL level that is intermediate, lying somewhere between a strong tendency and the absence of such behaviour. However, the specific level is influenced by a variety of factors.

In previous studies, domestic cats (*Felis catus*) were considered the only species that did not show CFL (Delgado *et al.* 2022). Researchers believe that cats are predatory animals, and their sit-and-wait predation is a low-energy and widespread hunting method (Williams *et al.* 2014). This foraging style of domestic cats makes them more inclined towards low energy feeding methods, showing lower CFL tendencies (Delgado *et al.* 2022). Animals such as domestic pigeons, mice (*Mus musculus*), and giraffes (*Giraffa camelopardalis*) belong within the category of foraging animals, requiring energy expenditure during the search for food (Inglis *et al.* 1997). They are more likely, therefore, to exhibit CFL tendencies under captive or caged conditions. Budgerigars in this study belong to exploratory foraging animals but did not exhibit strong CFL tendencies, demonstrating that foraging style can only partly explain the phenomenon of such behaviour.

The definition of CFL is the criterion used by researchers to determine whether animals exhibit CFL tendencies. Some researchers believe that when animals choose to make an effort to obtain food in more than 50% of cases, they are exhibiting CFL tendencies (Osborne 1977). Other researchers propose that according to the optimal foraging theory and standard learning theory, animals should choose free food over effortful food. Therefore, as long as animals select to make an effort to obtain food, they can be considered as exhibiting CFL tendencies (Inglis *et al.* 1997; McGowan *et al.* 2010; Ogura 2011; da Silva Vasconcellos *et al.* 2012). In this study, at the group level, the budgerigars’ first choices and the proportion of time spent at more challenging food containers did not differ significantly, results that were similar to those observed in African grey parrots (*Psittacus erithacus*) (Smith *et al.* 2022). Although they selected the more challenging food containers more than 50% of the time, half of the budgerigars did not exhibit strong contrafreeloading. Thus, we conclude that the CFL levels of budgerigars falls within an intermediate range, situated between a strong tendency and the absence of such behaviour.

In studying CFL, researchers may construct different forms of experimental apparatus relative to each species’ characteristics (such as body size, foraging method, food type, etc). For example, when studying starlings (*Sturnus vulgaris*), researchers provided food dishes covered with transparent and opaque plastic membranes for selection (Bean *et al.* 1999); when studying maned wolves, researchers set up food scattered areas and tray areas for selection (da Silva Vasconcellos *et al.* 2012); when studying domestic cats, researchers set up plates for direct access to food and food puzzles that required effort for food to be accessed (Delgado *et al.* 2022). Therefore, the inconsistent and diverse experimental settings may also affect animals’ choices. In this study, two food containers were set up in the birdcage for the budgerigars to select, the more challenging food container required the animals to move aside the wood-shavings covering the food (MC) or open a transparent plastic membrane before moving the wood-shavings to access the food (HC). In this way, animals may have their vision obstructed by the wood-shavings during feeding. However, since the food containers used in this study were transparent, the birds could see the food in both containers from a straight and downward slanting angle upon entering the feeding area of the cage. Therefore, we believed that the budgerigars’ vision would not affect the results of this study.

According to the theory of self-reinforcement mentioned above, if animals cannot achieve satisfaction in overcoming difficulties to obtain food, they will stop feeding, leading to the disappearance of CFL (Carder & Berkowitz 1970). In this study, the untrained budgerigars had a relatively low success rate (27%) in opening the highly challenging containers to access food, resulting in a loss of interest in HC food containers and a preference for less challenging food containers. In contrast, the pre-trained budgerigars had a relatively higher success rate (53%) in opening the highly challenging containers to access food, and they exhibited higher CFL tendencies than untrained budgerigars, supporting the theory of self-reinforcement.

According to the information primacy theory, hungry animals are more inclined towards free food and exhibit lower CFL levels (Singh 1970; Inglis & Ferguson 1986). In the ‘Free vs HC’ and ‘MC vs HC’ food container selection trials here, budgerigars with food deprivation, especially the HDP (8 h without food) showed lower CFL levels, similar to the results of red jungle fowl and starlings, indicating that animals exhibit lower CFL levels when hungry, supporting this theory (Inglis & Ferguson 1986; Lindqvist *et al.* 2002).

Besides, many other factors have been suggested as affecting CFL levels, such as age (McGowan *et al.* 2010), sex (Andrews *et al.* 2015), physical state (Sasson-Yenor & Powell 2019), food competition (Andrews *et al.* 2015), play opportunity (Smith *et al.* 2021), behavioural pathology (van Zeeland *et al.* 2023), and so on. The affecting factors from our study come from the five summarised explanations of Inglis *et al.* (1997) and apply to most animals that show CFL tendencies. Future studies might investigate the species-specific influencing factors on CFL levels.

Wild budgerigars are ground foragers that primarily seek cereals through extended searches. Contrary to their natural behaviour of CFL which offers adaptive advantages in the wild (Singh 1970; Inglis & Ferguson 1986), captive budgerigars are typically provided with abundant food, rendering foraging behaviours seemingly unnecessary. Consequently, caregivers often assume that simply supplying sufficient food is adequate. However, this study suggests that caged budgerigars maintain a certain level of internal motivation to explore and engage with their environment. The absence of opportunities to express these instincts may result in negative emotional consequences and health issues (Clubb & Mason 2003). Therefore, we would recommend that budgerigar caregivers offer appropriate challenges and choices for obtaining food within their living environment, thereby promoting opportunities for CFL behaviours to be expressed. Our results also have implications for the welfare of other caged or domesticated animals.

### Animal welfare implications

Budgerigars display certain tendencies for CFL. Budgerigars have bright feather colours, lively temperaments, and ease of domestication. They are highly favoured by bird lovers and kept as captive animals. Additionally, they are often used as experimental model animals due to their good learning abilities (Chen *et al.* 2019). The results of this study indicate that budgerigars tend to engage in some foraging behaviour rather than simple feeding activities with no food deprivation, having implications for the feeding of budgerigars by appropriately setting foraging environments to ensure their animal welfare. For example, budgerigars can be presented with two food containers: one that allows direct access to food, and another where the food is mixed with non-edible items (such as small stones or wood-shavings, as used in this experiment), requiring a degree of effort to locate and retrieve the food. This approach not only satisfies their basic feeding needs but also stimulates their natural inclination to explore and engage with their environment. Furthermore, the training experience, food deprivation, and effort required can affect their CFL levels, which lays a theoretical foundation for the subsequent study of vertebrate CFL.

## Supporting information

Tao et al. supplementary material Tao et al. supplementary material

## Supplementary material

The supplementary material for this article can be found at http://doi.org/10.1017/awf.2025.15.
