# Supplementary material for: Contrafreeloading and its influencing factors in budgerigars (Melopsittacus undulatus): Implications for their feeding and welfare
Source: Anim Welf. 2025 Mar 20;34:e21. doi: 10.1017/awf.2025.15 (PMC11949635; doi:10.1017/awf.2025.15)
Supplement: Tao et al. supplementary material [file S0962728625000156sup001.pdf]

Ethical Review of animal experiments

Editors,

The research project *Contrafreeloading and its influencing factors in Budgerigars* applied by Qi-Xin Zhang, Yue Tao, Yu-Ting Zhu, Hui Li, and Yong Zhu from our school involves the use of experimental animals. After review by the Institutional Committee for Animal Care and Use of Hefei Normal University, it is deemed that the animal experimental protocol and related materials of the research project meet the ethical requirements for animal welfare and animal experiments, and permission is granted to proceed with the research.

If there are any changes to the animal experimental protocol, the project leader is required to promptly notify the Institutional Committee for Animal Care and Use of Hefei Normal University for approval before implementation.

School of Biological and Food Engineering, Hefei Normal University

Institutional Committee for Animal Care and Use of Hefei Normal University

Scientific Research Administration of Hefei Normal University (with official seal)

December 11, 2023
